# Clinical outcomes of surgical and imatinib treatment for rectal gastrointestinal stromal tumours: retrospective cohort study

**DOI:** 10.1093/bjsopen/zrac067

**Published:** 2022-05-20

**Authors:** Shunsuke Tsukamoto, Yoshitaka Honma, Hirokazu Shoji, Hidekazu Hirano, Manabu Inoue, Yasuyuki Takamizawa, Konosuke Moritani, Jun Imaizumi, Yukihide Kanemitsu

**Affiliations:** 1 Department of Colorectal Surgery, National Cancer Center Hospital, Tokyo, Japan; 2 Department of Head and Neck, Esophageal Medical Oncology, National Cancer Center Hospital, Tokyo, Japan; 3 Department of Gastrointestinal Medical Oncology, National Cancer Center Hospital, Tokyo, Japan

## Abstract

**Background:**

Rectal gastrointestinal stromal tumours (GISTs) are rare and treated mainly by radical surgery. Although the importance of perioperative imatinib has been recognized, there are few reports on its outcomes.

**Method:**

Consecutive patients diagnosed with rectal GISTs between July 2008 and February 2021 were identified from a prospective database. Effects of perioperative imatinib were investigated, and surgical and survival outcomes were compared between neoadjuvant imatinib and upfront surgery.

**Results:**

34 patients meeting the inclusion criteria were identified. Compared with upfront surgery (n = 11), the neoadjuvant imatinib group (n = 23) had significantly larger tumours (median size 8.3 versus 2.5 cm; P = 0.01) and included a significantly greater proportion of high-risk patients according to the modified Fletcher classification (20/23 (87.0%) versus 6/11 (54.5%); P = 0.02). Comparing the operation planned based on imaging before neoadjuvant imatinib and the operation performed, there was an increase in sphincter-preserving surgery (4/23 (17.4%) to 11/23 (47.8%); P = 0.02), abdominoperineal resection 11/23 (47.8%) reduced to 7/23 (30.4%); P = 0.13) and total pelvic exenteration reduced from 8/23 (34.8%) to 5/23 (21.7%); P = 0.01). Tumours were downsized by a median of 30 per cent (range 0 per cent to −56 per cent; P =  0.01). During follow-up (median 42, range 5–131 months), there was no postoperative recurrence in 29 patients who received perioperative imatinib. One of the five patients who underwent surgery without neoadjuvant or adjuvant imatinib developed local recurrence.

**Conclusion:**

Treatment with imatinib for rectal GISTs seems to improve outcomes, and neoadjuvant imatinib increases the rate of sphincter-preserving surgery.

## Teaser

Effects of perioperative imatinib for rectal gastrointestinal stromal tumours (GISTs) were investigated, and surgical and survival outcomes were compared between neoadjuvant imatinib and upfront surgery. Multidisciplinary treatment with imatinib for rectal GISTs is very useful for improving oncological outcome, and neoadjuvant imatinib enables function-preserving surgery.

## Introduction

Gastrointestinal stromal tumours (GISTs) are the most common mesenchymal soft tissue tumour of the gastrointestinal tract but account for less than 1 per cent of all gastrointestinal tumours overall. Epidemiological studies consistently find the overall incidence of GISTs to be 6.5–15 per million population^1–3^. Although GISTs can originate from any site in the gastrointestinal tract, they most commonly arise in the stomach (50 per cent), proximal small intestine (35 per cent), and rarely in the rectum (5 per cent)^1,4^. Historically, surgery has been the mainstay of treatment for GISTs; however, surgery can be challenging in patients with rectal GISTs because the tumours are often large and the procedure must be performed in the anatomically narrow pelvic space. Rectal GISTs are often treated by radical surgery, including abdominoperineal resection, or total pelvic exenteration depending on the size of the tumour, invasion of other organs, and location. Postoperative quality of life is often poor because of organ or sphincter muscle loss leading to a permanent colostomy and impaired urogenital function^5,6^.

In recent years, postoperative imatinib therapy has improved overall survival and recurrence-free survival (RFS) in patients with GISTs and a high risk of recurrence after surgical resection^7,8^. There is evidence that preoperative imatinib therapy can reduce the size of locally advanced GISTs, thereby facilitating resection and lowering the risk of recurrence^9^. The benefits of neoadjuvant imatinib for operable rectal tumours include reduced morbidity, organ preservation, and a less-radical procedure, which may allow preservation of the sphincter muscles and avoid a permanent colostomy^10^. Several studies have demonstrated that preoperative use of imatinib improves surgical outcomes and highlighted the importance of a multidisciplinary approach to optimize patient outcomes in the imatinib era. Before the advent of imatinib, the goal of radical surgery was to achieve a microscopically margin-negative (R0) resection^11,12^; however, the risk of recurrence after microscopically margin-positive (R1) resection in the imatinib era has not been adequately evaluated. The numbers of cases of studies of neoadjuvant imatinib in GIST have been small, in the range of only 5–22 per study, because of the rarity of this tumour^8,11–17^. Therefore, there is no clear evidence that imatinib facilitates organ preservation or improves prognosis. The aims of this single-centre study were to evaluate the clinical profile of patients with rectal GISTs and to determine the effectiveness of neoadjuvant and adjuvant imatinib for allowing organ-preserving surgery and improving patient outcomes.

## Methods

### Patients

The data analysed in this study were obtained retrospectively from our prospectively maintained database at the National Cancer Center Hospital, Tokyo, Japan. Information has been compiled for all patients undergoing surgery for rectal GISTs since July 2008, when neoadjuvant imatinib was first used for rectal GIST at our institution. The cut-off date for this study was 8 August 2021. The study was approved by our institutional review board (2017-437). In all cases, the histological diagnosis was confirmed before treatment by core biopsy with positive immunostaining for CD117 (c-Kit). Patients with synchronous metastatic disease at the time of diagnosis were excluded. Data on patient demographics, tumour characteristics, radiological findings, surgical outcomes, preoperative and postoperative management, and recurrence were obtained from clinical records. Mutational analysis was performed in most patients from 2010 onwards and immunostaining for DOG1 status since 2013. The risk of aggressive behaviour of GISTs was determined by way of the modified Fletcher classification^18^.

All patients underwent CT of the chest, abdomen, and pelvis, and high-resolution MRI of the pelvis at the time of diagnosis. Patients who received neoadjuvant imatinib underwent CT and MRI after completion of neoadjuvant imatinib. Tumour size was measured before and after treatment by way of the measuring tool in the MRI software.

The indications for neoadjuvant imatinib were as follows: large tumours expected to have high surgical mortality and morbidity; invasion of adjacent organs and expected need for combined resection; invasion of the sphincter muscles and expected need for permanent colostomy after surgery; and Eastern Cooperative Oncology Group status 0–2. The starting dose of imatinib in neoadjuvant therapy was 400 mg/day. The final decision on the treatment plan was made at a multidisciplinary tumour board meeting consisting of surgeons, oncologists, radiologists, endoscopists, and pathologists. The duration of neoadjuvant imatinib was usually 6 months. CT evaluation was performed at 2-month intervals during neoadjuvant imatinib, and surgery could be performed at any time if the tumour stopped decreasing in size. If the tumour had clearly decreased in size by 6 months but a further decrease was expected, the duration of neoadjuvant imatinib could be extended. Adjuvant imatinib was given to patients in the upfront surgery group who were at high risk according to the modified Fletcher classification and to patients in the neoadjuvant group who considered to be at high risk according to the modified Fletcher classification based on tumour size on diagnostic imaging, or based on pathological examination of the resected specimen. For adjuvant therapy, imatinib was started at 400 mg/day and administered for 3 years, or dose adjusted in patients who required dose reduction during neoadjuvant treatment. The follow-up schedule was as follows: clinical examination and CT of chest, abdomen, and pelvis at 3-month intervals for 2 years and at 6-month intervals thereafter for at least 5 years after surgery.

Survival time was calculated from the date of surgery in the upfront surgery group and from the date of initiation of neoadjuvant imatinib in the neoadjuvant group. Local and distant recurrences were defined based on intraoperative, radiographical, and histological findings. Patients with gross tumour remnants intraoperatively (R2 resection) were excluded from the analysis of RFS.

### Statistical analysis

Differences between groups were examined with Mann–Whitney *U* and chi-squared tests. Survival curves were constructed with the Kaplan–Meier method and compared with the log rank test. All statistical analyses were performed with SPSS^®^ version 23.0 (IBM, Armonk, New York, USA). A *P* value lower than 0.05 was considered statistically significant.

## Results

### Patient and tumour characteristics

A total of 34 patients were diagnosed as having a primary rectal GIST during the 13-year study interval. Of these patients, 23 received neoadjuvant imatinib and 11 underwent upfront surgery. The clinicopathological characteristics of the two groups are compared in *Table 1*. The neoadjuvant group had a larger median tumour size at diagnosis (8.3 cm *versus* 2.5 cm; *P* < 0.01). The modified Fletcher classification showed a higher proportion of high-risk patients in the neoadjuvant group compared with the upfront surgery group (87.0 per cent *versus* 54.5 per cent; *P* = 0.02); the high-risk classification was based on pathological findings in the upfront surgery group and based on tumour size at diagnosis or pathological findings in the neoadjuvant group. All patients for whom immunohistochemical data were available were positive for CD34 and DOG1. Genomic mutations were measured in 14 patients, all of whom had mutations in exon 11 of *KIT*.

**Table 1:** Comparison of clinicopathological characteristics between the neoadjuvant imatinib group and the upfront surgery group

	Upfront surgery	Neoadjuvant imatinib	*P*
	(*n* = 11)	(*n* = 23)	
**Sex**			
Male	8	16	0.85
Female	3	7	
**Age at resection (years), median (range)**	61 (48–79)	63 (38–82)	0.86
**Tumour size at diagnosis (cm), median (range)**	2.5 (1.0–8.0)	8.3 (3.8–18.0)	<0.01
**Location** *			
≥5 cm	0	3	0.21
<5 cm	11	20	
**Mitotic count**			
0–5 per 50 HPF	5	7	0.31
6–10 per 50 HPF	2	4	
> 10 per 50 HPF	4	6	
Not performed	0	6	
**Modified Fletcher classification**			
Low risk	3	0	0.02
Intermediate risk	2	3	
High risk	6	20	
**CD34 status**			
Negative	0	0	0.58
Positive	10	22	
Not performed	1	1	
**DOG1 status**			
Negative	0	0	0.49
Positive	10	18	
Not performed	1	5	
**Mutation status**			
*KIT* exon 11	4	10	0.69
Not performed	7	13	

* Location was defined as the distance from the anal verge to the inferior margin of the tumour.

HPF, high-powered field.

Numbers are n unless otherwise stated.

### Response to neoadjuvant imatinib and surgery

**Fig. 1:**
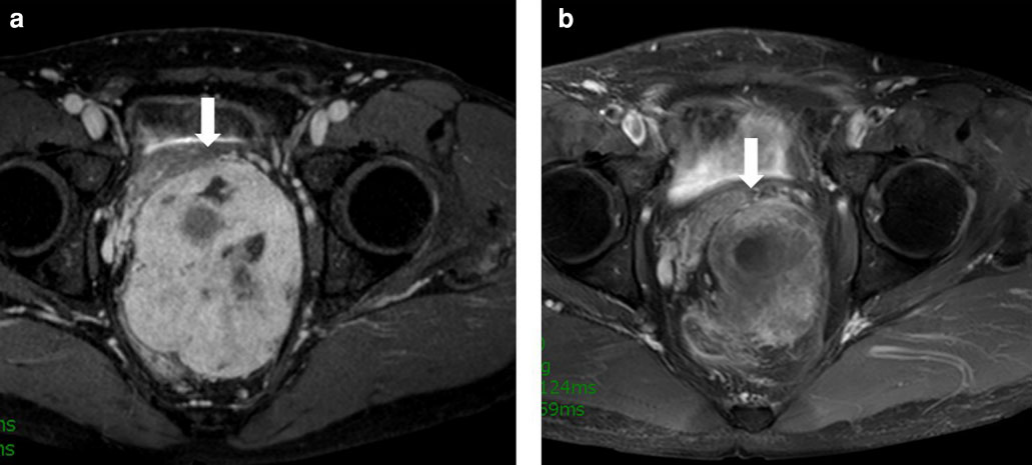
Rectal gastrointestinal stromal tumours

Neoadjuvant imatinib was administered for a median of 6 months (range 0–30); the median decrease in tumour size was −30 per cent (range 0 per cent to −56 per cent). In the neoadjuvant group, imatinib was discontinued after only 7 days in one patient who developed interstitial pneumonia, resulting in no change in tumour size. The other 22 patients completed neoadjuvant imatinib and achieved tumour downsizing. *Table 2* shows the preoperative data for the neoadjuvant group. Imaging showed that the proportion of patients with invasion of other organs decreased from 43.5 per cent to 26.1 per cent after neoadjuvant imatinib (*P* < 0.01). Based on the imaging findings before neoadjuvant imatinib, sphincter-saving surgery (low anterior resection or intersphincteric resection) was planned in 17.4 per cent of patients, abdominoperineal resection in 47.8 per cent, and total pelvic exenteration to achieve complete resection in 34.8 per cent; however, the decreases in tumour size and invasion of adjacent organs found on imaging after neoadjuvant imatinib resulted in significant changes to the preoperative plan, with sphincter-saving surgery being scheduled for 47.8 per cent of patients (*P* = 0.02), abdominoperineal resection for 30.4 per cent (*P* = 0.13), and total pelvic exenteration for 21.7 per cent (*P* < 0.01). Examples of pre- and post-treatment MRI for a patient with GIST are shown in *Fig. 1*. Surgery was performed according to the preoperative plan in all cases.

**a** Before imatinib treatment (maximum tumour diameter 10.0 cm). **b** After 11 months of treatment with imatinib (maximum tumour diameter 7.0 cm). Tumour shrinkage of 30 per cent was achieved after treatment compared with before imatinib. The border between the prostate and tumour was indistinct before imatinib administration, but became clear after imatinib administration (arrows). Total pelvic exenteration has initially been planned, but intersphincteric resection was ultimately performed.

**Table 2:** Preoperative evaluation before and after neoadjuvant imatinib

	Before neoadjuvant imatinib	After neoadjuvant imatinib	*P*
**Tumour size (cm), median (range)**	8.3 (3.8–18.0)	6.0 (2.3–11.0)	<0.01
**Invasion of other organs**			
No	13 (56.5)	17 (73.9)	<0.01
Yes	10 (43.5)	6 (26.1)	
Small intestine	1	0	
Prostate/seminal vesicles	7	5	
Vagina	2	1	
**Planned surgical procedure**			
Anterior resection/intersphincteric resection	4 (17.4)	11 (47.8)	0.02
Abdominoperineal resection	11 (47.8)	7 (30.4)	0.13
Total pelvic exenteration	8 (34.8)	5 (21.7)	<0.01

Numbers are n (%) unless otherwise stated.

### Surgical results and adjuvant imatinib therapy

**Fig. 2:**
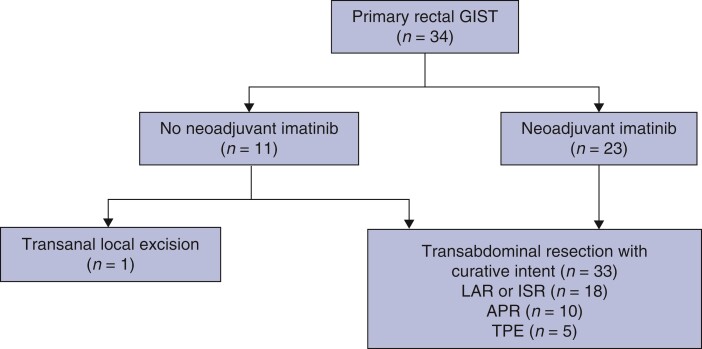
Overview of treatment pathways for patients with rectal gastrointestinal stromal tumours

APR, abdominoperineal resection; ISR, intersphincteric resection; LAR, low anterior resection; TPE, total pelvic exenteration.

The treatments performed during the study interval are summarized in *Fig. 2*. One patient in the upfront surgery group underwent transanal local excision and the remaining 33 patients underwent transabdominal resection (sphincter-saving surgery, *n* = 18; abdominoperineal resection, *n* = 10; total pelvic exenteration, *n* = 5). *Table 3* compares the pathological results between the two groups. Minimally invasive surgery was performed in 8 patients in the upfront surgery group and 7 patients in the neoadjuvant imatinib group, and open surgery was performed in 2 and 16 patients in each group respectively *(P* = 0.12). Median tumour size in the pathology specimen was significantly larger in the upfront surgery group (6.0 cm *versus* 3.5 cm). Pathological invasion of the prostate gland was found in two cases in the neoadjuvant therapy group. Complete resection was achieved in all patients in the upfront surgery group. In the neoadjuvant group, however, three patients had R1 resections, and R2 resections were observed in patients with intraoperative peritoneal dissemination or pelvic wall invasion. Of the 34 patients, 6 patients in the upfront surgery group had high risk according to the modified Fletcher classification at diagnosis, and 21 patients in the neoadjuvant therapy group, after exclusion of 2 patients with intraoperative tumour remnants who were considered to be candidates for adjuvant therapy. All the patients in the upfront surgery group received adjuvant imatinib but four patients in the neoadjuvant imatinib group did not receive adjuvant imatinib because of postoperative complications (*n* = 2) or patient refusal (*n* = 2). All three patients who underwent R1 resection received adjuvant imatinib. The two patients with R2 resection have been on continuous imatinib treatment for 27 and 36 months after surgery respectively.

**Table 3:** Comparison of pathological results between the two groups

	Upfront surgery	Neoadjuvant imatinib	*P*
	(*n* = 11)	(*n* = 23)	
**Pathological tumour size (cm), median (range)**	3.5 (0.9–8.0)	6.0 (2.5–15.0)	<0.01
**Pathological invasion of another organ**			
None	11	21	0.31
Prostate	0	2	
**Margin status**			
R0 resection	11	18	0.25
R1 resection	0	3	
R2 resection	0	2	

Numbers are n unless otherwise stated.

### Outcomes

Median follow-up duration after surgery was 42 months (range 5–131). Both the 3- and 5-year RFS rates were 100 per cent in the neoadjuvant imatinib group (excluding the two cases with R2 resection), and the respective rates were 100 per cent and 50 per cent in the upfront surgery group; the differences were statistically significant (*P* = 0.01). One patient in the upfront surgery group had local recurrence at 39 months after surgery. There were no cases of distant metastatic recurrence. The 5-year RFS for patients who received adjuvant imatinib and that for patients who underwent follow-up only (excluding the two cases with R2 resection) were significantly different between the adjuvant imatinib group and the follow-up-only group (100 per cent *versus* 66.7 per cent; *P* = 0.03). Neither of the two cases with R2 resection in the neoadjuvant group had postoperative recurrence. There were no deaths in either of the study groups during the study interval.

## Discussion

The tumours in patients who received neoadjuvant imatinib were larger and of higher grade than those in the upfront surgery group. The median size of the tumours treated with adjuvant imatinib in previous reports has ranged from 4.0 cm to 7.6 cm before treatment^8,12,14,19^, whereas the largest in this study was 8.3 cm. Patients deemed to be high risk according to the modified Fletcher classification and those who had received neoadjuvant chemotherapy were considered eligible for adjuvant imatinib. Despite these populations being considered to have high risk of recurrence, 29 patients who received imatinib perioperatively did not develop recurrence. A study from China comparing neoadjuvant imatinib with upfront surgery also showed favourable outcomes (5-year distant RFS at 97.8 per cent, 5-year disease-specific survival at 100 per cent) in the neoadjuvant group^20^. Another multicentre study from China demonstrated 3-year RFS at 95 per cent with patients receiving neoadjuvant imatinib^21^. This study demonstrated similar results to those reported elsewhere in Asia; however, the five patients who did not receive imatinib were at low risk clinically and pathologically, but one of them developed local recurrence. These results suggest that perioperative administration of imatinib for rectal GISTs may improve oncological outcomes.

Recurrence is a main problem after rectal GIST surgery, and a study from a large European cohort observed local recurrence in 15 per cent of cases^22^. Before the advent of imatinib, R1 resection of GIST was associated with high risk for local recurrence and R0 resection was considered the goal of radical surgery^11,12^; however, in a recent small study, Cavnar *et al*. reported that radical surgery with R1 resection is acceptable because local recurrence was absent in patients with R1 resection who were treated with adjuvant imatinib^14^. Similarly, in this study there was no postoperative recurrence in patients who received adjuvant imatinib, including three patients with R1 resection. Even with preoperative imatinib, many patients require resection of adjacent organs to achieve reliable R0 resection; however, if adjuvant imatinib can prevent recurrence with R1 resection, this might reduce the surgical resection margins required or extent of surgery, including removal of adjacent organs. The two patients with R2 resection who have been on continuous imatinib treatment after surgery have not had a recurrence to date. This suggests that imatinib might have a role in disease control in patients with R1/R2 resections, but further study is required.

The National Comprehensive Cancer Network recommends neoadjuvant systemic therapy for borderline resectable and oligometastatic or metastatic GISTs^23^. Neoadjuvant imatinib for operable rectal GIST offers the opportunity to downsize the tumour, thereby allowing organ preservation and a negative surgical margin^24–28^. Previous studies in the pre-imatinib era reported sphincter-preserving surgery rates of 28.5 per cent and 54.8 per cent^29,30^; however, reports on preoperative treatment with imatinib showed an increase in the rate of sphincter-preserving surgery in the range of 33.3–100 per cent^8,11,13,15–17,31^. Based on preoperative imaging, sphincter-preserving surgery was planned in only 17.4 per cent of patients but was actually performed in 47.8 per cent of those who received neoadjuvant imatinib. Furthermore, abdominoperineal resection was planned in 47.8 per cent of patients based on preoperative imaging but was performed in only 30.4 per cent of those in whom neoadjuvant imatinib was administered. Similarly, total pelvic exenteration, which was planned in 34.8 per cent of patients before preoperative treatment, was performed in only 21.7 per cent of those who received neoadjuvant imatinib. In this study, histologically invasive disease was confirmed in only two of five patients who underwent total pelvic exenteration due to preoperative invasion of adjacent organs. Therefore, even if preoperative imaging shows invasion, there is often no pathological invasion, meaning that preoperative imaging is not always correct; however, it is often not possible to identify the exact extent of tumour invasion during surgery, and the surgery is planned based on preoperative imaging. Therefore, preoperative therapy to ensure absence of invasion of adjacent organs is important to achieve organ preservation.

A major concern about neoadjuvant imatinib is that the tumour will progress if it is ineffective. The type of *KIT* mutation has been found to predict the response to imatinib^32–34^. Patients with mutations involving exon 11 in *KIT* have a better complete or partial response rate (63–83.5 per cent) than those with exon 9 mutations (25–47.8 per cent) or wild-type *KIT* (0–7 per cent) and therefore may benefit the most from imatinib therapy^33,35,36^. In this study, tumour shrinkage was achieved in all patients who were able to receive imatinib, except for one patient who developed interstitial pneumonia and could not complete preoperative therapy. All 10 patients in the neoadjuvant imatinib group who underwent genetic testing had mutations in exon 11, which may have led to the favourable tumour shrinkage rate. In nine retrospective studies of patients with rectal GIST treated with neoadjuvant imatinib, only one patient developed progressive disease^8,11–17,31^. This suggests a low rate of disease progression in patients with rectal GISTs who receive preoperative imatinib. Therefore, it may be preferable to administer imatinib first to determine the response regardless of the size of the tumour before proceeding to surgery; however, it may be necessary to check for *KIT* mutations before treatment, as there may be a small number of tumours with *PDGFR*α mutations or wild-type that do not respond well to imatinib.

The standard dose of imatinib is reported to be 400 mg/day^8^. However, an analysis showed that high-dose imatinib (800 mg/day) demonstrated longer progression-free survival compared with the standard dose for patients with metastatic GISTs with exon 9 mutations^37^. However, in a retrospective series of adjuvant therapy for exon 9-mutated GISTs, high-dose imatinib did not achieve better survival outcomes compared with the standard dose^38^. In the present study, all patients received the standard dose because the national health insurance system in Japan allows administration of only up to 400 mg/day, and the median reduction in tumour size was 30 per cent. While the standard dose may be sufficient for many cases with exon 11 mutations, such as those included in the present study, higher doses may be necessary for cases of progressive disease with exon 9 mutations. Prospective studies will be needed to determine the indicated doses for these patients. The consensus on the optimal duration of neoadjuvant imatinib is that imatinib should be continued until the maximal response is observed^39^, which may take at least 6 months according to the National Comprehensive Cancer Network guidelines^23^. In this study, we used 6 months as a guide but left the final decision regarding shrinkage of the tumour to the discretion of the physician; however, decisions varied from physician to physician, such that the median duration of neoadjuvant imatinib ranged from 6 months to 30 months. The appropriate duration of neoadjuvant imatinib will need to be clarified in future clinical trials.

This study has several limitations. First, the data were obtained from a single centre, and although the number of cases is larger than in previous reports, the actual number was still small. For a disease as rare as GIST, it is necessary to establish a multicentre case registration system to clarify its clinicopathological features in detail. Second, mutational status was not examined in many of the older cohort, which meant that the association with genes could not be adequately investigated. Mitotic counts were not performed in all cases. The follow-up duration was relatively short, especially for the most recent patients who were followed up for less than 1 year. Further studies with longer follow-up periods are required.

## Data Availability

The datasets generated during and/or analysed during this study are available from the corresponding author on reasonable request.
